# Arginine to Glutamine Variant in Olfactomedin Like 3 (*OLFML3*) Is a Candidate for Severe Goniodysgenesis and Glaucoma in the Border Collie Dog Breed

**DOI:** 10.1534/g3.118.200944

**Published:** 2019-02-01

**Authors:** Carys A. Pugh, Lindsay L. Farrell, Ailsa J. Carlisle, Stephen J. Bush, Adam Ewing, Violeta Trejo-Reveles, Oswald Matika, Arne de Kloet, Caitlin Walsh, Stephen C. Bishop, James G. D. Prendergast, Joe Rainger, Jeffrey J. Schoenebeck, Kim M. Summers

**Affiliations:** * The Roslin Institute and Royal (Dick) School of Veterinary Studies, Easter Bush, EH25 9RG, United Kingdom; † Nuffield Department of Clinical Medicine, University of Oxford, John Radcliffe Hospital, Oxford, OX3 9DU, United Kingdom; ‡ Mater Research Institute-University of Queensland, Translational Research Institute, Brisbane, Qld 4102, Australia; § Animal Genetics, 1336 Timberlane Rd, Tallahassee, FL 32312

**Keywords:** glaucoma, goniodysgenesis, olfactomedin like 3, Border Collie, eye development

## Abstract

Goniodysgenesis is a developmental abnormality of the anterior chamber of the eye. It is generally considered to be congenital in dogs (*Canis lupus familiaris*), and has been associated with glaucoma and blindness. Goniodysgenesis and early-onset glaucoma initially emerged in Border Collies in Australia in the late 1990s and have subsequently been found in this breed in Europe and the USA. The objective of the present study was to determine the genetic basis of goniodysgenesis in Border Collies. Clinical diagnosis was based on results of examinations by veterinary ophthalmologists of affected and unaffected dogs from eleven different countries. Genotyping using the Illumina high density canine single nucleotide variant genotyping chip was used to identify a candidate genetic region. There was a highly significant peak of association over chromosome 17, with a *p*-value of 2 × 10^−13^. Expression profiles and evolutionary conservation of candidate genes were assessed using public databases. Whole genome sequences of three dogs with glaucoma, three severely affected by goniodysgenesis and three unaffected dogs identified a missense variant in the olfactomedin like 3 (*OLFML3*) gene in all six affected animals. This was homozygous for the risk allele in all nine cases with glaucoma and 12 of 14 other severely affected animals. Of 67 reportedly unaffected animals, only one was homozygous for this variant (offspring of parents both with goniodysgenesis who were also homozygous for the variant). Analysis of pedigree information was consistent with an autosomal recessive mode of inheritance for severe goniodysgenesis (potentially leading to glaucoma) in this breed. The identification of a candidate genetic region and putative causative variant will aid breeders to reduce the frequency of goniodysgenesis and the risk of glaucoma in the Border Collie population.

Companion animals including cats, dogs and horses, suffer from a range of diseases, with both genetic and environmental aetiologies. The breed barrier created by registration requirements and breeding practices seeking to maximize compliance of animals with breed specifications, result in increased levels of matings between relatives (Asher *et al.* 2009). This means that purebred companion animals are particularly likely to suffer from recessive genetic conditions with large effect sizes. Understanding the genetic factors underlying these diseases is important to improve welfare within breeds. The strong linkage disequilibrium in such inbred populations indicates that small numbers of pedigree animals can be used to identify genetic regions of interest, making them excellent genetic models if the same disease affects humans.

In response to the identification of putative causal variants, breeding strategies can be put in place to reduce the prevalence of conditions that impact on animal welfare, especially when a genetic test can be developed. However, the success of these approaches depends on understanding the mode of inheritance and level of genetic contribution to the disease, and ideally on identifying a causative variant that can be used to develop a test for genetic status.

Primary glaucoma is a condition in which increased ocular pressure damages the retinal ganglion, leading to blindness (reviewed in Fricker *et al.* (2016)). In dogs (*Canis lupus familiaris*) it can be preceded by goniodysgenesis (also known as mesodermal dysgenesis), a developmental abnormality of the eye characterized by narrowing or closure of the iridocorneal angle through which the aqueous humor drains. This is associated with alterations in the structure of the pectinate ligament which traverses the drainage angle, called pectinate ligament dysplasia (PLD) (Plummer *et al.* 2013) (more properly pectinate ligament abnormality (Oliver *et al.* 2017b)). In the Leonberger dog breed, goniodysgenesis has been associated with an increased risk of developing primary closed angle glaucoma (Fricker *et al.* 2016) and in Flat-Coated Retrievers, development of glaucoma is positively and significantly related to the severity of goniodysgenesis (Wood *et al.* 1998). Goniodysgenesis is generally considered to be congenital in dogs, although its subsequent progression varies among breeds. Severity is thought to increase with age in Leonbergers (Fricker *et al.* 2016) and Flat-Coated Retrievers (Pearl *et al.* 2015) and was shown to be strongly associated with age in Dandie Dinmont Terriers, Basset Hounds, Flat-Coated Retrievers, Hungarian Vizslas and Golden Retrievers (Oliver *et al.* 2016; Oliver *et al.* 2017a). However, goniodysgenesis remained stable in Samoyeds (Ekesten and Narfstrom 1991) and was not associated with age in a small study of Border Collies (Oliver *et al.* 2017a).

Anecdotal reports suggest that sudden onset glaucoma leading to blindness with bilateral loss of eyes was seen initially in young Border Collies in Australia in the late 1990s. A similar, sudden onset glaucoma in young animals subsequently emerged in the UK Border Collie population, often in dogs that were related to the affected Australian Border Collies. As breeders recognized that there was a potential issue, they began to voluntarily track the results of gonioscopies and reports of glaucoma in an online database (Border Collie Goniodysgenesis and Glaucoma Database; https://bc-glaucomadatabase.synthasite.com/). A number of dogs in the USA and Europe have now also been diagnosed with severe goniodysgenesis and glaucoma (Border Collie Goniodysgenesis and Glaucoma Database). Goniodysgenesis in Border Collies was added to the Schedule B list of “Conditions Under Investigation” in the British Veterinary Association (BVA) Eye Scheme (http://www.bva.co.uk/Canine-Health-Schemes/Eye-scheme/) and examination of pedigrees indicated it was highly prevalent in some Border collie lineages (see Anadune Border collie database: http://www.anadune.com/). In a recent study (Oliver *et al.* 2017a), 11 of 102 Border Collies (10.8%) were reported to have moderate or severe PLD (associated with goniodysgenesis). Seven (6.9%) were mildly affected. The Border Collie goniodysgenesis and Glaucoma Database lists 110 dogs which have been diagnosed as affected with goniodysgenesis, but only 12 who have developed glaucoma. It is not known why a proportion of dogs diagnosed with severe goniodysgenesis go on to develop glaucoma but breeders report that some have lived for as long as 15 years and remain free of glaucoma. There is an apparent association with a small number of popular sires, and a genetic etiology is strongly suspected in Border Collies, but the heritability and mode of inheritance of the condition is currently unknown.

The aim of the current study was to perform a genome-wide analysis to find genetic regions that are associated with severe goniodysgenesis and glaucoma in the Border Collie and identify candidate variants that might be responsible for this condition.

## Materials and Methods

Full details of the Materials and Methods, including details of publicly available data, are given in File S1.

### Ethical approval

All studies were approved by the Veterinary Ethical Review Committee of the University of Edinburgh (approval number VERC 2012-8) and the Animal Ethics Committee of the University of Queensland (approval number ANFRA/MRI-UQ/565/17).

### Ascertainment of clinical status and pedigree

Goniodysgenesis status for individuals in the study was obtained from a database of gonioscopy test results in Border Collies (Border Collie Goniodysgenesis and Glaucoma Database), or from official test certificates submitted by the owners. Goniodysgenesis was diagnosed by veterinary ophthalmologists (such as members of the BVA Eye Panel in the UK or the equivalent certifying body in other countries) who examined the eyes with gonioscopy and assessed the status of the drainage angle and the pectinate ligament fibers that span it. Depending on the year and country of testing, results may have been given as “clinically unaffected/affected”, or there may have been more information about the extent of goniodysgenesis. Some reports specified both whether the angle was structurally narrow and the extent of PLD. Other results allocated a grade to the level of goniodysgenesis. An early grading scheme in the UK had Grade 1 being very mild and Grade 5 being severely affected. Currently a new grading scheme is being evaluated in the UK, with Grade 1 being mild and Grade 3 being severely affected (British Veterinary Association Eye Scheme). Since we had results in multiple formats, each report was manually assessed to assign clinical status to each subject. Only individuals whose eyes had been examined using gonioscopy were included. Three cases with comorbidity (cataract, autoimmune disease) and one case where genotype results were inconsistent with official parentage were excluded from all analyses.

Breeding records and pedigree data were obtained from Anadune Border Collie database, which contains over 226,700 registered Border Collie dogs worldwide, from the Border Collie Pedigree Database (http://db.bordercollie.ru), with 20,217 dogs, and from the breeders’ websites.

A genome wide association study (GWAS) was undertaken using the more extreme phenotypes. As such, we defined cases as dogs that had goniodysgenesis of grades four or five under the early grading scheme, dogs whose goniodysgenesis was described as “severe”, those where the veterinary ophthalmologist report indicated PLD over more than 70% of the iridocorneal angle and those that developed glaucoma following a diagnosis of goniodysgenesis. Controls were dogs that were assessed as clear or clinically unaffected based on at least one gonioscopy examination. Previous work did not show age-related progression of goniodysgenesis in Border Collies (Oliver *et al.* 2017a) so the controls were those with a normal gonioscopy result, regardless of age.

### Genetic analysis

DNA samples were from buccal cells collected by owners and occasionally from blood. Full details of collection and extraction procedures are given in File S1. Genotyping used the Illumina 173K CanineHD Whole-Genome Genotyping Bead Chip (Illumina, San Diego, CA, USA) and was performed by Edinburgh Genomics, University of Edinburgh, UK. Results were filtered in PLINK v1.07 (Purcell *et al.* 2007) to remove individuals that had more than 10% of missing genotypes and markers that had rates of genotyping <0.95, had minor allele frequency <0.05 in this population or deviated from the Hardy-Weinberg equilibrium in the controls with *p* value of less than 0.0001. Only the autosomes were considered.

The GWAS was performed in GEMMA v0.94.1 (Zhou and Stephens 2012) using a linear mixed model approach, controlling for relatedness using a SNV-based relatedness matrix as a random effect. The significance threshold was 4.8 × 10^−7^ calculated using an uncorrected P value of 0.05 adjusted for testing on 104,141 segregating SNVs (Bonferroni correction).

Whole genome sequencing (WGS) at 30X coverage of DNA from three dogs with glaucoma, three with severe goniodysgenesis and three unaffected animals was performed using the Illumina HiSeq X platform. Library insert sizes were 450 bp and paired reads of 150 bp were sequenced. Library preparation and sequencing was performed by Edinburgh Genomics. Reads were processed by standard procedures, detailed in File S1, and mapped to the CanFam3.1 reference genome. The significance of variants was assessed using PolyPhen-2 (http://genetics.bwh.harvard.edu/pph2), Mutation Taster (http://www.mutationtaster.org), Provean (http://provean.jcvi.org) and SIFT (scores obtained from Provean analysis). Structural variants were ascertained with DELLY 0.7.7 (Rausch *et al.* 2012) and GRIDSS 2.0.1 (Cameron *et al.* 2017).

### Transcriptomic analysis of candidate region genes

A time course of mouse eye development from embryonic day 12 to adult was examined using the FANTOM5 database (FANTOM Consortium and the RIKEN PMI and CLST (DGT) *et al.* 2014; Arner *et al.* 2015; Summers and Hume 2017) (FANTOM5 Browser, http://fantom.gsc.riken.jp/zenbu). FANTOM5 data were also used to obtain expression levels for human eye-related samples (Table S1). Transcriptomic data for the developing chicken eye were generated for another project by RNA sequencing methodology (J. Rainger *et al.*, unpublished data). Details are given in File S1. Microarray-based expression data for a range of tissues in human and mouse were from BioGPS (http://biogps.org) as were RNA sequencing data for chicken tissues. Microarray expression data for immune cell types was derived from the Immunological Genome Project (https://www.immgen.org/).

### Analysis of canine OLFML3 and CDK2AP1 genes

The region of the canine *OLFML3* gene containing the candidate causative variant was amplified by the polymerase chain reaction and the amplicon sequenced by chain termination sequencing (Australian Genome Research Facility (AGRF), Brisbane, Australia or Edinburgh Genomics, University of Edinburgh, UK). The change associated with the variant in the *CDK2AP1* gene created a cutting site for the restriction enzyme *Hin*f1. DNA was amplified by the polymerase chain reaction and treated with *Hin*f1 to identify the uncut (wild type) and cut (risk) alleles. The genotypes of a number of samples of all genotypes were validated using chain termination sequencing (AGRF). Full details of primers and protocols are given in File S1. Sequences for analysis of nucleotide and amino acid conservation across species were downloaded from Ensembl (http://www.ensembl.org) and alignment was performed using Clustal Omega (http://www.ebi.ac.uk/Tools/msa/clustalo/).

### Data availability

The SNV data used in the GWAS are available via the University of Edinburgh data repository at http://dx.doi.org/10.7488/ds/2426. BAM files for the WGS are available via the European Nucleotide Archive, accession numbers ERS2643230 and ERS2643240-7. The variant calls for *OLFML3* and *CDK2AP1* are available in Table S3. Supplemental material available at Figshare: https://doi.org/10.25387/g3.7414073.

## Results

### Genome wide association analysis

**Figure 1:**
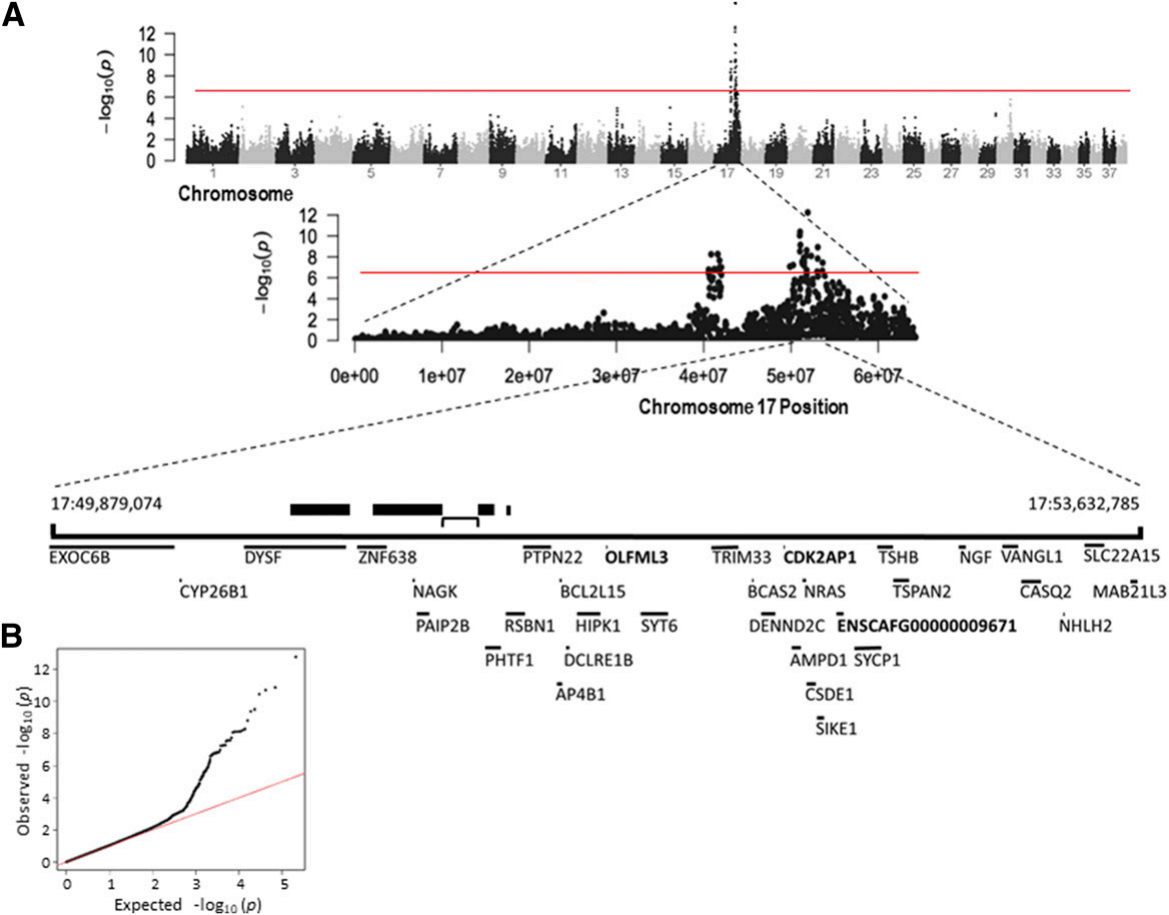
Genome wide association with severe goniodysgenesis and glaucoma A. Manhattan plot for the canine genome; chromosome 17 is shown in more detail and genes in the region are shown below. Thick bars show known large structural variants and the bracket indicates the location of the break in synteny between dog and other mammals. Red line shows the Bonferroni significance level (4.8 × 10^−7^). Genes in bold are those where a coding sequence variant was detected. B. QQ-plot for the GWAS analysis. The lambda value is 1.07.

**Figure 2:**
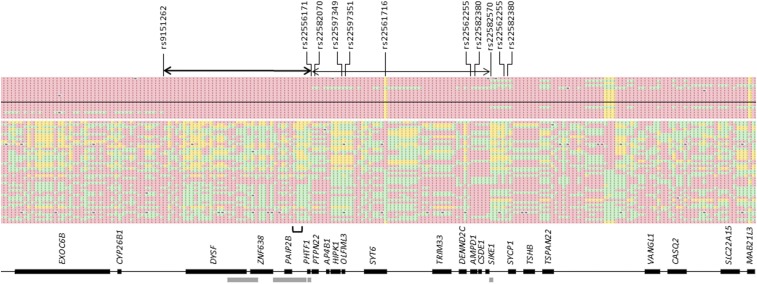
Homozygosity within the region of significant SNVs, CanFam3.1 17:49,879,074-53,632,785. Each row is a DNA sample and each column shows a variant. Only variants that were segregating in this population are shown. Red and yellow indicate homozygosity for the two alleles and green shows heterozygosity. The upper block shows animals with severe goniodysgenesis (above black line) and glaucoma (below black line); the lower block shows those passed as clinically unaffected at gonioscopy testing. The most significant SNV rs22561716 is yellow in the cases (homozygous for the risk-associated allele); one clinically unaffected animal was homozygous for this allele and one severely affected animal was heterozygous. SNVs rs9151262 and rs22582070 flank the block of homozygosity (heavy black arrow). rs22582070 and rs22582570 flank a second block where all cases except one were homozygous (light black arrow). SNV rs22556171 indicates the point at which the genotypes of full siblings BC6108 (affected) and BC6113 (unaffected) become the same. Genes underlying the SNVs are indicated by black bars; the relative proportions are different from Figure 1 reflecting the differing density of markers across the region. There were no segregating markers for *RSBN1*, *BCL2L15*, *DCLRE1B*, *BCAS2*, *CDK2AP1*, *NRAS*, *ENSCAFG00000009671*, *NGF* or *NHLH2*. SNVs rs22597349 and rs22597351 flank the *OLFML3* gene. SNVs rs22562255 and rs22582380 flank the *CDK2AP1* gene. SNVs rs22562255 and rs22582380 flank *ENSCAFG00000009671*. Gray bars show known large structural variants and the bracket indicates the location of the break in synteny between dog and other mammals.

To identify genetic regions associated with goniodysgenesis in this breed, we performed a GWAS on 17 cases (8 female, 9 male) with goniodysgenesis described as “severe” (N = 4), Grade 4 or 5 by the earlier grading scheme (N = 1 and 5 respectively), or goniodysgenesis followed by glaucoma (N = 7). Controls were 42 dogs (26 female, 16 male) who had been passed as “clinically unaffected” at veterinary ophthalmology testing. A strong association of goniodysgenesis with a region of chromosome 17 was found (Figure 1A). The lowest *p*-value was for SNV rs22561716 at 17:51,919,221 (*P* = 2 × 10^−13^). Genotypes for this variant are shown in Table 1. SNVs with significant *p*-values (*P* ≤ 4.8 × 10^−7^) were in a region of 3.8 Mbp between 17: 49,879,074 and 17:53,632,785 (Figure 2). This region contains a break in synteny where the mapped genes are continuous in the dog and on different chromosomes in other mammals; the break occurs between *PAIB2B*/*NAGK* and *PHTF1* (indicated by a bracket in Figures 1 and 2). The region also contains a number of large structural variants including deletions and inversions (the largest are indicated by thick bars in Figures 1 and 2) (Decker *et al.* 2015).

**Table 1:** Genotype frequencies for the most significant SNV, rs22561716, in severely affected and unaffected dogs from the GWAS analysis

Genotype for rs22561716	TT	TG	GG	TOTAL
Clinical status				
**Glaucoma**	7	0	0	7
**Severe goniodysgenesis**	9	1	0	10
**Unaffected**	1	21	20	42

There was a block of shared homozygosity in affected animals from 17:50,895,797 (distal to rs9159262) to 17:51,666,793 (proximal to rs22582070) (indicated in Figure 2). This did not include the SNV with the lowest *p*-value (rs22561716 at 17:51,919,221), which lay just beyond an apparent recombination breakpoint in one animal affected with Grade 5 goniodysgenesis. The region was further delineated because the full siblings BC6108 (affected) and BC6113 (unaffected) were homozygous up to rs22556171. Therefore the region where all cases and no controls were homozygous for the risk allele extended from 17:51,510,172 (distal to rs22556171) to 17:51,666,973 (proximal to rs22582070) as shown in Figure 2. Within the genomic region identified by the association analysis there were a number of coding genes (Figure 1A), although none was known to be involved in abnormalities of eye development or development of glaucoma in animals or humans. Several had strong expression in the developing mouse eye (Figure S1). In particular there were three genes within the minimal overlap region (*PHTF1*, *RSBN1*, *PTPN22*). *Ptpn22* had negligible expression in the developing mouse eye. Expression of *Phtf1* was moderate in the embryonic eye and declined after birth. Expression of *Rsbn1* was also moderate in the mouse eye, peaking in the early neonatal period (Figure S1). These genes were therefore considered to be candidates for the pathological variant in this condition.

### Variant detection by whole genome sequencing

We then obtained the whole genome sequence of this region for three dogs with glaucoma, three severely affected with goniodysgenesis but without glaucoma and three unaffected individuals, who were members of four nuclear families (Figure S2). All were distantly related through multiple lineages in the extended pedigree. Relatives were chosen to highlight variants that were different between affected and unaffected family members (Lander and Botstein 1987).

There were no structural variants where all cases were the same and different from all controls. Several deletions were noted, but in general all animals carried these deletions (Table S2). This is consistent with the small number of segregating variants in this region, indicating a high level of homo- or hemizygosity.

Our initial scan of the block of shared homozygosity (heavy arrow, Figure 2) revealed ten variants for which all cases were homozygous for the non-reference allele and controls were heterozygous or homozygous for another allele (green fill in Table S2). There were six annotated protein coding genes in this region but there were no coding sequence or frameshift variants. Six of the variants were intronic (two in *DYSF*, one in *PIAP2B* and three in *RSBN1*) and four were intragenic. Three of the intronic and one of the intragenic variants were located in the critical overlap region between 17:51,510,172 and 17:51,666,793 (dark green fill in Table S2). The intronic variants in *RSBN1* all affect a tetranucleotide repeat (TAAA_14_) in the middle of the intron. The intronic variants in *DYSF* affect a tetranucleotide repeat (TAAA_11_) and a string of 11 T residues. There was no evidence that any of these variants might be pathological.

Since there were no potentially detrimental variants in the region of shared homozygosity, we considered DNA changes beyond the apparent recombination breakpoint (where one Grade 5 affected animal became heterozygous) up to 17:52,484,845 (rs22582570) where three additional cases became heterozygous (light arrow in Figure 2 and blue fill in Table S2). This region included the most significant SNV. The other sixteen severely affected and glaucoma cases were homozygous in this region (Figure 2). Between the putative breakpoint at 17:51,666,793 and 17:52,484,845 there were 15 annotated coding genes. The region contained 114 variants where the six sequenced cases were all homozygous and the controls were heterozygous or homozygous for another allele. Two variants were missense and one was synonymous. There were 19 intron variants and the remainder were intergenic or in the up- or downstream gene regions (Table S2).

**Figure 3:**
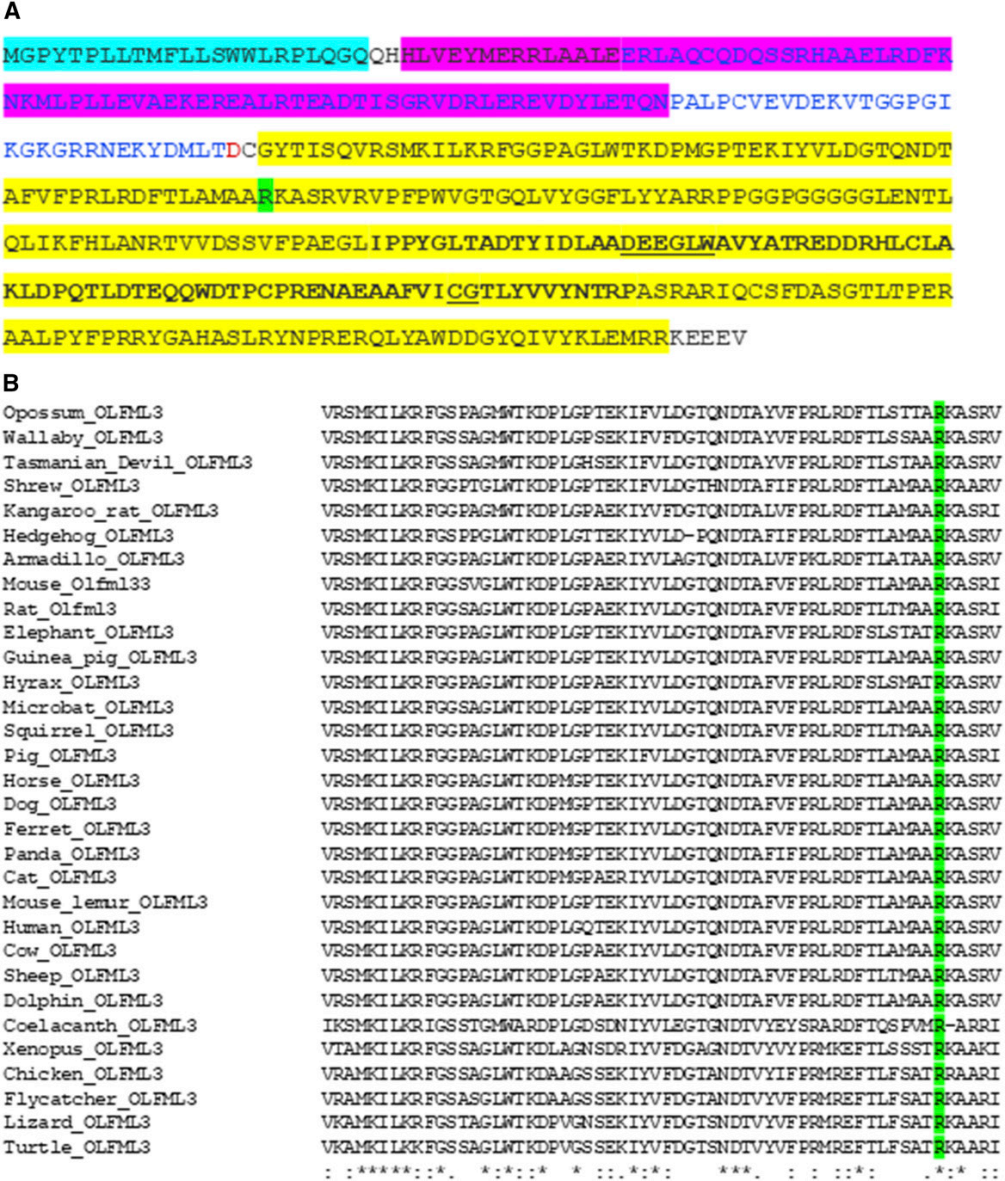
Conservation of *OLFML3* predicted amino acid sequence A. Amino acid sequence of the canine OLFML3 protein. Functional domains are highlighted: blue – signal peptide; pink – coiled coil domain; yellow – olfactomedin domain (Zeng *et al.* 2005; Rodríguez-Sánchez *et al.* 2013). Within the yellow region, bold lettering shows the core OLF region. Green shows the arginine at position 197. B. Conservation of arginine 197 (green) in OLFML3 of vertebrates. Arginine is found at the equivalent position in all mammal, bird and reptile species for which there is an annotated *OLFML3* gene, and in coelacanth but not in other fish. The region around this arginine is also highly conserved.

A missense variant was found in the olfactomedin like 3 (*OLFML3*) gene, at position 17:51,786,924 (CanFam3.1, Ensembl), between rs22597349 and rs22597351 and close to the most significant SNV (Figure 2). The variant is ENSCAFT00000014855.3:c.590G>A with the predicted change of arginine (codon CGG) to glutamine (codon CAG) at position 197 (ENSCAFP00000013747.3:p.Arg197Gln). This arginine is in the olfactomedin (OLF) domain (Rodríguez-Sánchez *et al.* 2013; Zeng *et al.* 2005) (Figure 3A) and was found to be highly conserved throughout mammals, birds and reptiles (Figure 3B). In humans, the equivalent variant (ENST00000320334.4:c.587G>A) corresponds with rs377336789 (SNV database, https://www.ncbi.nlm.nih.gov/snp/) and was seen in four out of 120,920 alleles with a minor allele frequency of 3.3 X 10^−5^; no homozygotes were seen (Exome Aggregation Consortium, http://exac.broadinstitute.org; (Lek *et al.* 2016)). The variant effect prediction program PolyPhen-2 gave a score of 0.888, considered “possibly damaging”. Another effect prediction program, Mutation Taster, considered the variant to be “disease causing” with potential changes to the protein features and a possible splice site change. In contrast, Provean found that the variant was likely neutral (score: 0.03). The SIFT score for the human variant was 0.429, categorized as “tolerated.”

The A allele was not seen in 213 genotypes from 12 dog breeds (not including Border Collies) and four wolf populations from the Multiple Dog Genome Project (High quality variant calls from multiple dog genome project - Run1, https://www.ebi.ac.uk/eva/?eva-study=PRJEB24066). Four of 34 Border Collies from the Dog Biomedical Variant Database Consortium (DBVDC) were heterozygous (giving an allele frequency of 0.055 in this breed) but the A allele was not seen in 504 genotypes of other breeds. Notably, it was not present in 51 Leonbergers, three Basset Hounds, eight Golden Retrievers, one Flat Coated Retriever, three Welsh Springer Spaniels or one Dandie Dinmont Terrier, all breeds reported to have a high prevalence of goniodysgenesis (Table S3). In addition, 350 dogs recorded as Border Collies but of unknown clinical status were genotyped by Animal Genetics (Talahassie, Florida, USA) via allele-specific PCR run on an ABI 3730 machine. There were 2 homozygotes and 29 heterozygotes among these 350 Border Collies, giving an allele frequency of 0.05, consistent with the smaller sample from the Consortium.

Taken together the evidence suggested that the *OLFML3* variant was likely to have a mild to moderate effect on the protein. The greatly increased frequency in the cases is strong support while the conflicting computation and predictive data provides weaker support (Richards *et al.* 2015). Segregation with severe goniodysgenesis/glaucoma was observed within families (as seen in the nuclear families from the WGS and also in additional families where the *OLFML3* genotype was established), providing strong support for likely pathogenesis (Richards *et al.* 2015). We therefore examined this gene further.

### OLFML3 genotypes of severely affected and unaffected Border Collies

The results for the *OLFML3* variant ENSCAFT00000014855.3:c.590G>A in glaucoma, severe goniodysgenesis and unaffected dogs are shown in Table 2. The individual who was heterozygous for the region based on SNV results was also AG at *OLFML3*. This animal had been diagnosed with Grade 5 goniodysgenesis but did not develop glaucoma over a 15-year lifespan. In addition, one unaffected dog was AA genotype. This animal was the offspring of a severely affected (grade 5) father and a mother diagnosed as affected (with no grade). Both parents were also AA. Under some testing schemes animals could pass with a degree of goniodysgenesis (Fricker *et al.* 2016; Oliver *et al.* 2017b) which may be the case with this dog. In general, the genotype for *OLFML3* segregated with that for rs22561716, indicating strong linkage disequilibrium between them. However, an unaffected mother and son were homozygous for G at *OLFML3* but heterozygous at rs22561716 (shown by the different numbers of heterozygotes and wild type homozygotes in Table 1 and Table 2). In addition, two unaffected individuals were homozygous for several SNVs either side of *OLFML3* but heterozygous for the *OLFML3* variant and rs22561716.

**Table 2:** Genotype frequencies for *OLFML3* mutation c.590G>A in severely affected and unaffected dogs

Genotype for *OLFML3*	AA	AG	GG	TOTAL
Clinical status				
***GWAS dogs*^1^**				
**Glaucoma**	7	0	0	7
**Severe goniodysgenesis**	9	1	0	10
**Unaffected**	1	19	22	42
***Replication dogs***				
**Glaucoma**	2	0	0	2
**Severe goniodysgenesis**	3	1	0	4
**Unaffected**	0	17	8	25
**TOTALS**				
**Glaucoma**	9	0	0	9
**Severe goniodysgenesis**	12	2	0	14
**Unaffected**	1	36	30	67

1 Dogs that were included in the genome wide analysis (GWAS dogs) are shown separately from dogs in the replication set that were only tested for *OLFML3*.

To validate these results, we genotyped 31 additional Border Collies that had had gonioscopy investigations: two with glaucoma (both female), four with severe goniodysgenesis (all female) and 25 unaffected (10 male, 15 female). The genotypes of this replication set of dogs are shown in Table 2 and are consistent with the results suggesting that severe goniodysgenesis/glaucoma was associated with homozygosity for the A allele of *OLFML3*.

### OLFML3 genotypes in mildly affected and uncategorized affected Border Collies

Our initial analysis compared dogs affected with severe goniodysgenesis or glaucoma with dogs passed as clinically unaffected at gonioscopy examination. Because of changes in the reporting scheme in the UK and differences among countries, there were a number of equivocal diagnoses. These were dogs for which either the degree of goniodysgenesis was reported as mild (Grade 1 under the former scheme or stated as mild or marginal on the report; N = 9; five female, four male), moderate (Grade 2 – 3 under the old scheme, or stated as moderate or between 25 and 70% blocked on the report; N = 9; eight female, one male), or the dogs were diagnosed as affected with goniodysgenesis with no description given (N = 15; nine female, six male). To examine whether *OLFML3* was involved in these cases we genotyped these 33 dogs. The results are shown in Table 3. All three genotypes were found among these cases.

**Table 3:** Genotype frequencies for *OLFML3* mutation c.590G>A in mild, moderate and uncharacterized affected dogs

Genotype for *OLFML3*	AA	AG	GG	TOTAL
Clinical status				
**Mild**	3	3	3	9
**Moderate**	4	2	3	9
**Affected, no grade**	7	3	5	15
**TOTALS**	14	8	11	33

### Expression of OLFML3 in the eye

**Figure 4:**
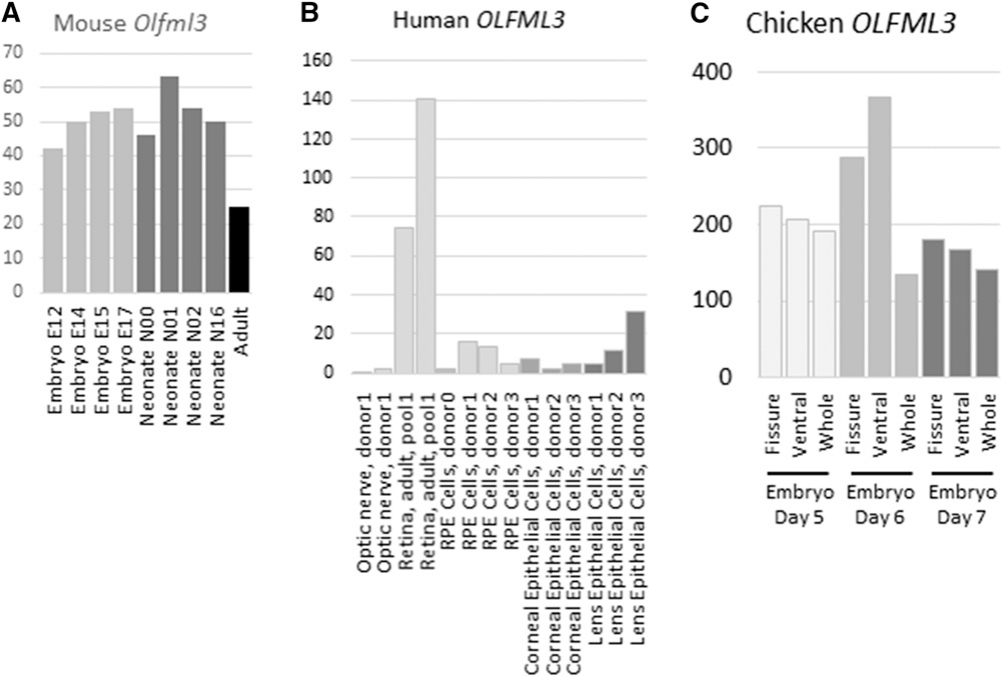
Expression of *OLFML3* genes in eye regions A. Expression of *Olfml3* in developing mouse eyeball. Results are based on CAGE and were downloaded from the FANTOM5 database (http://fantom.gsc.riken.jp/5/tet). Y axix shows RLE normalized values. Light gray – embryonic samples; dark gray – neonatal samples; black – adult sample. B. Expression of *OLFML3* in human eye tissues and cells. Results are based on CAGE and were downloaded from the FANTOM5 database. Y axis shows RLE normalized values. Light gray – optic nerve and retina; mid gray – cornea; dark gray – lens. C. Expression of *OLFML3* in the developing chick eye. Results are based on RNA sequencing (J. Rainger *et al*., unpublished data). Y axis shows transcripts per million (TPM) values. Light gray – embryonic day 5; mid gray – embryonic day 6; dark gray – embryonic day 7.

In expression data generated by the FANTOM5 project, the *Olfml3* gene was strongly expressed in mouse eyeball at embryonic and neonatal stages and declined in the adult sample (Figure 4A). FANTOM5 data also showed that the gene was expressed in human lens, corneal epithelium and retina (Figure 4B), although the highest expression was in amniotic and placental epithelial cells and in mesenchymal stem cells (not shown). *OLFML3* was also expressed in the developing chick eye, with highest expression at embryonic day 6 (Figure 4C). In mouse microarray data (mouse probeset 1448475_at; available at BioGPS) expression of *Olfml3* was highest in osteoblasts undergoing calcification, but it was also found in eyecup, iris, ciliary bodies and lens (Figure S3A). There was negligible expression in immune system cells, except for macrophages, osteoclasts and particularly microglia, which had expression comparable to lens and eyecup. Strong expression in microglia was also seen in the Immunological Genome data set (Figure S3B). Human microarray data also available through BioGPS showed strongest expression of *OLFML3* in adipocytes, uterus and retina, the only eye tissue represented (human probeset 218162_at) (Figure S3C). RNA sequencing data compiled on BioGPS revealed expression in many samples from the chicken, notably the whole embryo and connective tissues and the cornea of the adult (Figure 3D). Other OLF family members were also expressed in the developing mouse eyeball with a similar pattern to *Olfml3*, peaking in the early neonatal period and declining in the adult eye (Figure S4). In contrast, the myocilin gene (encoding a related protein which is mutated in human open angle glaucoma) had negligible expression in embryo and early neonate but increased expression at neonatal day 16 and in adult eyeball (Figure S4).

### Other genetic variants in the region of interest

Two other potentially damaging variants were found in the region of the most significant SNV. One was a missense variant in the cell cycle gene *CDK2AP1* (ENSCAFT00000015099.1:c.236C>T; CanFam3.1 17:52,363,576). This would result in the substitution of leucine for proline at amino acid 79 (ENSCAFP00000041716.1:p.Pro79Leu). The gene has been mapped to three canine chromosomes on the UCSC browser (UCSC Genome Browser, https://genome.ucsc.edu); a location on canine chromosome 26 is syntenic with the mapped position of the gene in human and mouse, so there is some uncertainty about whether the region on chromosome 17 represents a functional gene, a pseudogene or an incorrect genome assembly. Using the equivalent human DNA change (matching the canine gene on chromosome 26), PolyPhen-2 gave a score of 0.995, considered “probably damaging”. Mutation Taster considered the variant to be “disease causing” with potential changes to the protein features. Provean also found that the variant was likely deleterious (score: -8.44). In contrast the SIFT score for the human variant was 0.121, categorized as “tolerated”. This variant has been annotated as rs850595562 with an allele frequency of 0.05 in 218 canines (12 dog breeds and four wolf populations), including three homozygotes (High quality variant calls from multiple dog genome project - Run1). There were no Border Collies in this dataset and none of the breeds included has been reported to have a predisposition to goniodysgenesis. The variant was found in 46 of 550 dogs in the DBVDC set, including eight homozygotes (Table S3). The allele frequency was quite high in Border Collies (12 heterozygotes and one homozygote in 34 dogs) but also high in other breeds such the Dogue de Bordeau (three homozygotes and two heterozygotes in six dogs) and the Cane Corso (two homozygotes and two heterozygotes in five dogs). In contrast most other breeds known to be susceptible to goniodysgenesis and glaucoma were uniformly homozygous for the wild type allele, except for one Golden Retriever which was heterozygous. Targeted disruption of the gene in mouse (equivalent to the chromosome 26 gene in dog) resulted in a high incidence of embryonic lethality (Kim *et al.* 2009). The presence of multiple versions of this gene in the canine genome may explain the apparent lack of phenotype associated with this variant in the dog and suggests the possibility that the chromosome 17 gene is non-functional. The majority of the dogs with severe gonidysgenesis or glaucoma were homozygous for the risk allele (Table S4). However, two unaffected dogs were also homozygous for the risk allele and two goniodysgenesis affected dogs were heterozygous. The variant was in strong linkage disequilibrium with the most significant SNV, but there were a number of examples with apparent recombinant haplotypes. This variant seemed unlikely to be causal for severe goniodysgenesis in Border Collies.

A potentially damaging variant was found beyond the region of greatest homozygosity (distal to rs22582570, flanked by rs22562255 and rs22582380). This was a frameshift variant involving deletion of the second nucleotide of exon 7 of *ENSCAFG00000009671* (CanFam3.1 17:52,526,443-52,536,071), encoding a putative protein orthologous to mouse nuclear receptor NR1H5. The *Nr1h5* gene has negligible expression in the developing mouse eye and is represented by a pseudogene in the human genome. All genotypes for the variant were found in the Multiple Dog Genome Project. All sequenced severe goniodysgenesis and glaucoma dogs in our study carried the reference sequence while controls were heterozygous for this and the deletion and it was unlikely that this would be the cause of their severe goniodysgenesis and glaucoma.

## Discussion

In a number of dog breeds development of glaucoma has been associated with the presence of goniodysgenesis. In the Flat-Coated Retriever, the likelihood of glaucoma was positively and significantly related to the severity of goniodysgenesis (Wood *et al.* 1998) and in the Leonberger five unilateral glaucoma-affected individuals all had a high grade of goniodysgenesis in the contralateral eye (Fricker *et al.* 2016). An abnormal drainage angle is considered typical of some breeds, with reports of up to 81% of individuals showing evidence of goniodysgenesis (Ekesten and Narfstrom 1991; Kato *et al.* 2006). However, goniodysgenesis does not necessarily proceed to glaucoma. For example, one study found that 25% of Samoyeds had some level of PLD, but only 3% had developed glaucoma (Ekesten and Narfstrom 1991). The highest prevalence of glaucoma (all forms) reported in any single breed is only 5.52% (in the American Cocker Spaniel) (Gelatt and MacKay 2004).

An increasing incidence of glaucoma in young animals of the Border Collie breed has been anecdotally reported by breeders over the last 15 years. Inspection of available Border Collie pedigrees revealed that there were three common ancestors in both the sire and dam lineages for all cases of glaucoma on the Border Collie Goniodysgenesis and Glaucoma Database (N = 12, including two pairs of full siblings both affected with glaucoma), as well as additional cases not on the database (N = 4). These common ancestors dated back to the late 1970s and early 1980s and were found in multiple ancestral lineages for the glaucoma cases. All the glaucoma cases had been diagnosed with goniodysgenesis and subsequently had one or both eyes removed. Consistent with results in other breeds, we found some Border Collies who lived entire lifetimes with apparently severe goniodysgenesis without developing glaucoma. This included two popular sires who reached the age of 15 years without a diagnosis of glaucoma, although they had produced puppies who did develop glaucoma; three dogs with severe goniodysgenesis had produced a total of six offspring with glaucoma. It also included a full sibling of two dogs with glaucoma. Although severe goniodysgenesis is important in the progression to glaucoma, it is not the sole cause. The etiology may involve a genetic predisposition to goniodysgenesis combined with genetic, environmental or random factors influencing development of glaucoma.

The diagnosis of goniodysgenesis was made by veterinary ophthalmologists (such as members of the BVA Eye Panel in the UK or the equivalent certifying body in other countries) who performed gonioscopies to assess the status of the drainage angle and the pectinate ligament fibers that cross it. Since the diagnoses were made by a number of different clinicians over a period of years, there may be considerable noise in the phenotype. An initial eye scheme developed by the BVA in association with the Kennel Club of the United Kingdom involved assessing whether the dog had no eye abnormality (a pass), or some abnormality graded from one to five according to the extent of PLD and the width of the iridocorneal angle, in increasing levels of severity. Since there was a high degree of inter-observer variability in assigning a grade, the scheme was subsequently amended such that the dog would be reported as either clinically unaffected or affected. Dogs that were considered unaffected could have a drainage angle that was abnormal to some extent (“mild” goniodysgenesis; see information available at https://www.bva.co.uk/Canine-Health-Schemes/Eye-scheme/). The binary scheme was expected to have a higher level of inter-observer agreement. This was confirmed in a recent study of Leonberger dogs (Fricker *et al.* 2016) where there was a high correlation in proportion affected with goniodysgenesis between a prospective study where examinations were performed by a single specialist (2012-2014; 18% affected) and a retrospective study of BVA eye scheme certificates (2009-2014; 22% affected). In a study of PLD in Welsh Springer Spaniels, there was a good correlation between two examiners in determining unaffected eyes, although there was considerable variability between examiners in assigning the degree of PLD (Oliver *et al.* 2017b). This variability in categorizing the extent of goniodysgenesis means that individuals with “clinically unaffected” phenotype can have mild PLD or angle narrowing (Fricker *et al.* 2016; Oliver *et al.* 2017b). For this reason, we focused our genome-wide analysis on severely affected dogs including several that had gone on to develop glaucoma.

Using dogs with severe goniodysgenesis or goniodysgenesis followed by glaucoma as cases and clinically unaffected dogs as controls, we found a strong peak of association on chromosome 17, with a stretch of shared homozygosity of about 1 Mbp. However, whole genome sequencing of three control, three glaucoma and three severe goniodysgenesis dogs showed that there were few variants in this region where the cases were all of the same genotype and different from all controls. Variants in this region were all either intronic or intragenic and there were no coding sequence variants. Intronic variants all affected short microsatellite repeat regions. Dogs have a high level of unstable microsatellites in coding or non-coding regions, sometimes associated with disease (Fondon and Garner 2004; Forman *et al.* 2015). However the repeat length changes in *DYSF* and *RSBN1* were small (± 1-3 repeat units) in the middle of introns and were found in a number of breeds in the Multiple Dog Genome Project. They seemed unlikely to be pathogenic. The number of segregating SNVs in this region was low and all animals carried a number of deletions across the region.

Slightly distal to this region but where 16 of 17 cases were still homozygous in the GWAS, and closer to the most significant SNV, we detected two missense variants. One, in *CDK2AP1*, was felt unlikely to be causative. Homozygotes for the risk allele were found in a range of breeds in which goniodygsenesis has not been reported and the allele frequency was relatively high across all breeds. We also found two unaffected dogs in our study who were homozygous for this allele. The presence of three orthologs of *CDK2AP1* in the canine genome suggested that there was redundancy, potentially with the gene in the region of interest being non-functional. A frameshift mutation in *ENSCAFG00000009671* was also segregating in Border Collies, but all the cases were homozygous for the reference allele, making it unlikely that this was the cause of the condition.

We also detected a missense variant, ENSCAFP00000013747.3:p.Arg197Gln, in the *OLFML3* gene. We propose that this variant is a candidate for severe goniodysgenesis potentially leading to glaucoma in the Border Collie, based on the presence of the variant only in this breed, the probability that there is some deleterious effect from the amino acid change, strong conservation of the sequence in vertebrates including non-mammalian species and the segregation of the variant with clinical status in canine families. In addition, spatial and temporal expression patterns of the gene are consistent with a role in eye development.

Although this gene has not been implicated in goniodysgenesis or glaucoma in humans, it is strongly expressed in tissues of the anterior segment of the human and baboon eye including lens, iris, sclera and trabecular meshwork (Rodríguez-Sánchez *et al.* 2013; Tomarev and Nakaya 2009) and in the developing mouse eye (Ikeya *et al.* 2005). The *Olfml3* gene was expressed throughout mouse eye development (Figure 4A) and during chick eye development (Figure 4C). In mouse, inactivation of the *Olfml3* gene by insertion of a *Lac* cassette resulted in viable and fertile homozygotes (Ikeya *et al.* 2005), consistent with the relatively mild selective disadvantage to dogs with goniodysgenesis. Expression has also been observed in human tissues of mesenchymal origin such as bone and adipose. Olfactomedin like 3 is an extracellular matrix protein that has been implicated in the epithelial to mesenchyme transition in cancer (Schmitz *et al.* 2015), in keeping with its mesenchymal expression and suggesting that abnormality of this protein could be associated with the formation or retention of abnormal sheets of mesenchyme in the drainage angle. Olfactomedin like 3 is also proangiogenic (Miljkovic-Licina *et al.* 2012) and interacts with a member of the bone morphogenic protein (BMP) family, BMP4. Since BMPs including BMP4 are involved in eye development (see (Pillai-Kastoori *et al.* 2015) for a review), this may indicate the pathway for olfactomedin like 3 action.

In addition, expression of *OLFML3* is characteristic of microglia in humans (Butovsky *et al.* 2014; Gosselin *et al.* 2017) and mice (Figure S3; see also Neidert *et al.* (2018) and data from Grabert *et al.* (2016)), although it is not strongly expressed by other macrophage lineages. Genetic manipulation resulting in absence of microglia is associated with loss of *Olfml3* expression in mice (R. Rojo *et al.*, unpublished data). The retina contains microglia which are important for retinal health (Hume *et al.* 1983; Karlstetter *et al.* 2010), and they could be one source of *OLFML3* mRNA in the canine eye. Opening of the iridocorneal angle involves considerable remodelling of the mesenchymal tissue within the angle, including thinning and extending of the pectinate ligament and opening the trabecular meshwork (Trejo-Reveles *et al.* 2018). Microglia may contribute to this process as eye development progresses, by phagocytosing apoptotic cells and debris from the remodelling. Abnormality of olfactomedin like 3 in the microglia of the eye could interfere with this function and therefore disrupt the opening of the iridocorneal angle and formation of the proper drainage channels.

Olfactomedin like 3 is a member of a large family characterized by the olfactomedin (OLF) domain (Zeng *et al.* 2005; Anholt 2014), with several members expressed in eye structures. In particular, the glucocorticoid-inducible family member myocilin (*MYOC* gene) has been implicated in dominant open angle glaucoma in humans (Stone *et al.* 1997) and is strongly expressed in the trabecular meshwork of the drainage angle (Tamm *et al.* 1999; Tomarev *et al.* 2003). As with *Olfml3* mutants, there is no gross phenotype in *Myoc* knockout mice (Kim *et al.* 2001). Increased expression of *Myoc* mRNA resulted in a reduction in *Olfml3* mRNA (Paper *et al.* 2008) in a mouse model, suggesting that there is an inverse association between these two OLF proteins, consistent with the expression profiles in the developing mouse eye (Figure S4) where *Olfml3* declined in the adult while *Myoc* rose. *In vitro* experiments indicated that myocilin interacts with olfactomedin 3 (also known as optimedin, encoded by the *OLFM3* gene) which was co-expressed with myocilin in the eye (Torrado *et al.* 2002). A variant in another OLF family member, *OLFM2*, has been associated with human open angle glaucoma in a small number of Japanese patients (rs779032127; p.Arg144Gln) (Funayama *et al.* 2006) and identified as contributing to eye development (Holt *et al.* 2017). These other OLF family members may compensate for olfactomedin like 3 abnormalities, or may interact with olfactomedin like 3 in the development of the eye. Hence genetic variation in these family members may modify the impact of the *OLFML3* variant in the Border Collies, explaining the variable phenotype of homozygotes and heterozygotes carrying the risk variant.

A limitation to the hypothesis that the ENSCAFP00000013747.3:p.Arg197Gln variant in Border Collies is a risk allele for the presence of severe goniodysgenesis predisposing to glaucoma in homozygous animals is the observation that the variant lies outside the region of shared homozygosity (although within the GWAS peak) and two animals with apparently severe glaucoma were heterozygous. We have also seen some heterozygous animals with mild to moderate phenotypes (although none with glaucoma), but most heterozygotes have been passed as clinically unaffected at gonioscopy testing. As discussed by Miano *et al.* (2000), one explanation would be segregation of a second allele where compound heterozygosity also causes goniodysgenesis. Wakeling *et al.* (2018) showed that the predictive power of homozygous regions is reduced where samples have greater than 8% homozygosity overall; a high degree of homozygosity was noted in the Border Collies in this study.

In addition, there may be variants at modifier loci that can compensate for or exacerbate the effect of the *OLFML3* variant. In particular, the presence of many OLF proteins in the eye and the mild phenotype associated with relatively broadly expressed gene family members suggests redundancy for at least some functions. It is also possible that inadequate compensation for the putative effects of the ENSCAFP00000013747.3:p.Arg197Gln variant may be responsible for the progression from severe goniodysgenesis to glaucoma.

There were too few glaucoma cases in our study to allow identification of loci involved in progression from goniodysgenesis to glaucoma in the Border Collie and ambiguous phenotyping complicates the investigation of mild goniodysgenesis. The genetic relationship between the mild forms and the more severe forms is unknown, but the *OLFML3* variant described here does not appear to be associated with most cases of mild goniodysgenesis indicating that genetic predisposition to mild goniodysgenesis may be independent of the findings of this study. Although testing for the *OLFML3* variant (whether it is causative or in linkage disequilibrium with the causative DNA variant) would allow breeders to select against homozygotes to decrease the prevalence of the risk genotype for severe goniodysgenesis, it is important not to reduce the breeding pool too much because of the risk of other recessive conditions resulting from homozygosity for alleles that are identical by descent. Although we need to understand why some goniodysgenesis cases progressed to glaucoma at a young age and some do not, before we can make firm recommendations to the breeders of Border Collies worldwide, we suggest that following DNA testing dogs of all genotypes could be used provided at least one partner in the mating is homozygous for the wild type allele. This would ensure that no homozygotes for the risk allele were produced. We would strongly advocate that efforts be made to maintain the breadth of the gene pool by not avoiding the use of animals that carry this variant. As discussed previously (Farrell *et al.* 2015), strong selection away from variants serves to narrow genetic variability and can give rise to future health problems. All breeding decisions should be made with this, and the wider health of the breed, in mind.
